# Ubiquitination and Degradation of the Hominoid-Specific Oncoprotein TBC1D3 Is Mediated by CUL7 E3 Ligase

**DOI:** 10.1371/journal.pone.0046485

**Published:** 2012-09-27

**Authors:** Chen Kong, Dmitri Samovski, Priya Srikanth, Marisa J. Wainszelbaum, Audra J. Charron, Jialiu Liu, Jeffrey J. Lange, Pin-I Chen, Zhen-Qiang Pan, Xiong Su, Philip D. Stahl

**Affiliations:** 1 Department of Cell Biology and Physiology, Washington University School of Medicine, St. Louis, Missouri, United States of America; 2 Department of Oncological Sciences, Icahn Medical Institute, New York, New York, United States of America; 3 Department of Internal Medicine, Center for Human Nutrition Washington University School of Medicine, St. Louis, Missouri, United States of America; German Cancer Research Center, Germany

## Abstract

Expression of the hominoid-specific TBC1D3 oncoprotein enhances growth factor receptor signaling and subsequently promotes cellular proliferation and survival. Here we report that TBC1D3 is degraded in response to growth factor signaling, suggesting that TBC1D3 expression is regulated by a growth factor-driven negative feedback loop. To gain a better understanding of how TBC1D3 is regulated, we studied the effects of growth factor receptor signaling on TBC1D3 post-translational processing and turnover. Using a yeast two-hybrid screen, we identified CUL7, the scaffolding subunit of the CUL7 E3 ligase complex, as a TBC1D3-interacting protein. We show that CUL7 E3 ligase ubiquitinates TBC1D3 in response to serum stimulation. Moreover, TBC1D3 recruits F-box 8 (Fbw8), the substrate recognition domain of CUL7 E3 ligase, in pull-down experiments and in an *in vitro* assay. Importantly, alkaline phosphatase treatment of TBC1D3 suppresses its ability to recruit Fbw8, indicating that TBC1D3 phosphorylation is critical for its ubiquitination and degradation. We conclude that serum- and growth factor-stimulated TBC1D3 ubiquitination and degradation are regulated by its interaction with CUL7-Fbw8.

## Introduction

*TBC1D3* is a hominoid-specific oncogene encoded by a cluster of eight paralogs on chromosome 17 [1]. It is over-represented in several human cancer genomes, including prostate, breast [2], pancreatic [3] and gall bladder tumors [4], as well as in myelodysplastic syndrome [5]. Moreover, exogenously expressed TBC1D3 is able to transform mouse cells [6], [7] and induce tumor formation in nude mice [2], suggesting a causative link between TBC1D3 expression and tumorigenesis.

The precise biological function of TBC1D3 and the role it plays in tumor development and progression are still under investigation. TBC1D3 was initially shown to contain a TBC domain, a domain normally associated with the Rab GTPase Activating Proteins (GAPs) [2]. However, TBC1D3 had only modest GAP activity [2], which was later explained by the absence of an “arginine finger” required in the catalytic center of a GAP [8]. The TBC domain was subsequently shown to be a Rab-binding domain [8].

Several reports have implicated TBC1D3 in regulation of receptor-mediated growth factor (GF) signaling. Two reports have explored the connection between TBC1D3 and epidermal growth factor receptor (EGFR) trafficking and signaling. Our previous study [9] showed that TBC1D3 expression enhanced the activation of Ras in response to epidermal growth factor (EGF), thus promoting cell proliferation. Moreover, we documented [9] that TBC1D3 expression suppressed recruitment of Cbl, an E3 ubiquitin ligase, resulting in decreased ubiquitination and delayed degradation of EGFR. A second study showed that through enhanced signaling by the EGFR, TBC1D3 expression contributed to optimal propagation of EGFR-mediated signaling and stimulated macropinocytosis [8].

Finally, our understanding of the impact of TBC1D3 expression on cell signaling was expanded by studies on insulin receptor (IR) signaling. We documented that through an interaction with protein phosphatase 2A (PP2A), TBC1D3 inactivates p70 S6 kinase, thereby leading to delayed degradation of insulin receptor substrate-1 (IRS-1) and sustained insulin/insulin-like growth factor 1 (IGF-1) signaling [10]. Overall, these findings suggested that TBC1D3 expression increases the activation of several GF-stimulated signaling pathways, thus enhancing cell growth and proliferation.

In the current study, we explored the effect of GF stimulation on TBC1D3 protein turnover. We discovered that stimulation with GFs triggered ubiquitination and proteasomal degradation of TBC1D3, suggesting a negative feedback loop that suppresses the effect of TBC1D3 on receptor signaling.

To further elucidate the molecular mechanisms of GF-induced ubiquitination and degradation of TBC1D3, we performed a yeast two-hybrid screen to identify TBC1D3-interacting partners. The screen identified Cullin 7 (CUL7), a molecular scaffold protein, as a TBC1D3-binding partner. CUL7 is a member of Cullin family of proteins that facilitate the assembly of Cullin-RING E3 ubiquitin ligase complexes (CRLs). CRLs are multi-protein complexes that are composed of a scaffold backbone linked through an adapter protein (such as Skp1, S-phase kinase associated protein1) to a substrate recognition protein (such as an F-box protein) [11]. At the C-termini the scaffold contains a Cullin domain that interacts with the ROC1/Rbx1 RING finger protein. Many members of the Cullin family, e.g., CUL1, interact with most, if not all members of the F-box family [12]. However, CUL7 is unique among Cullin family E3 ligases in that it assembles with a sole F-box recognition subunit called Fbw8 (F-box and WD repeat domain containing 8), which in turn recognizes a limited number of substrates for ubiquitination.

In the work described here we confirmed the interaction between CUL7 and TBC1D3 both *in vitro* and *in vivo*, by several experimental approaches. Furthermore, we identified the CUL7-Fbw8 complex as a key effector of GF-induced TBC1D3 ubiquitination and degradation. The results reported in this study document a novel mechanism for regulation of TBC1D3 protein turnover. We postulate that GF-induced ubiquitination of TBC1D3 controls its degradation, thus keeping the effect of TBC1D3 on cell signaling and proliferation at bay. Dysregulation of the molecular mechanisms that control the degradation of TBC1D3 in various tissues would potentially precipitate the development and progression of tumors in humans.

## Experimental Procedures

### Yeast Two-Hybrid Screening for TBC1D3-interacting proteins

Yeast two-hybrid screening was performed using the Matchmaker 3 system (Clontech/Takara). Briefly, AH109 yeast was transformed with the plasmid pGBK-T7 encoding the GAL4 DNA-binding domain fused in frame to full-length TBC1D3. Transformants were mated with Y187 yeast containing the plasmid pACT2, encoding cDNAs from a human fetal brain library fused to the GAL4 transcriptional activation domain. Diploids were plated onto quadruple drop-out nutritional selection plates and colonies put through two rounds of isolation. Plasmids were rescued, prey-containing pACT2 plasmids amplified in *E. coli*, and the inserts were sequenced. Interaction between TBC1D3 and prey proteins was verified by co-immunoprecipitation of epitope-tagged, *in vitro* transcribed/translated proteins (TNT T7, Promega) after subcloning partial or full-length prey coding-sequences into pGAD-T7.

### *In vitro* transcription/translation assay

Plasmid inserts encoding putative TBC1D3 binding partners were transcribed and translated *in vitro* using a coupled, T7 polymerase-driven reaction with ^35^S-methionine. A polyclonal anti-TBC1D3 antibody was bound to adsorb TBC1D3 purified from a baculoviral expression system or to bovine serum albumin as a control. The synthesized radiolabeled proteins were then incubated with the TBC1D3-Sepharose using native conditions. Following several washes to eliminate non-specific binding, the samples were eluted in SDS sample buffer, analyzed using SDS-PAGE and TBC1D3-binding proteins were visualized by autoradiography.

### Plasmids and reagents

The cDNA for full-length TBC1D3 was amplified by PCR and ligated into EcoRI/NotI sites of a pCMV-Myc vector (Invitrogen). Full-length HA-tagged CUL7 and truncated CUL7 mutant consisting of amino acid residues 268–1698 were kindly provided by Dr. J. DeCaprio (Dept. of Medical Oncology, Dana-Farber Cancer Institute). pCR3.1-Myc-Fbw8 and GST-Fbw8-Skp1 were from Dr. Zhen-Qiang Pan (Dept. of Oncological Science, Mount Sinai School of Medicine). Anti-HA and Myc antibodies were from Santa Cruz; anti-tubulin, GAPDH and CUL7 (monoclonal) were from Sigma; anti-ubiquitin was from Invitrogen; 2C7 monoclonal anti-TBC1D3 antibody was generated by the Washington University Hybridoma Center with the last 50 amino acids of TBC1D3 as antigen.

### Cell culture and transfections

HeLa cells (American Type Culture Collection, Manassas, VA) were grown in Dulbecco's modified Eagle's medium (DMEM) (Hyclone) supplemented with 10% fetal bovine serum and penicillin/streptomycin. All transfections were carried out using Lipofectamine 2000 (Invitrogen) according to manufacturer's instructions. Approximately 18 h after transfection, cells were processed as described for each experiment. Small interfering RNAs (siRNAs) were prepared with a Silencer siRNA Construction Kit (Ambion). The sequence of siRNA used for CUL7, 5′-AACUGCCAUGUCUACAAGAAG-3′; a universal negative control siRNA was from Sigma.

### Immunoblot analysis

Whole cell lysates in lysis buffer (50 mM Tris-HCl, pH 7.5, 100 mM NaCl, 1% Triton X-100, 10% glycerol, 1 mM EDTA, and protease inhibitor cocktail supplemented with 10 mM NaF and 1 mM Na_3_VO_4_) were separated by SDS-PAGE and transferred to nitrocellulose membranes (Schleicher & Schuell, Germany). The membranes were blocked in TBST (100 mM NaCl, 10 mM Tris-HCl, pH 7.5, 0.1% Tween 20) containing 5% non-fat milk and incubated with primary antibodies in 2% BSA/TBST overnight at 4°C or 2 h at room temperature, followed by incubation with HRP-conjugated goat anti-rabbit or anti-mouse IgG (Jackson ImmunoResearch, West Grove, PA) and analyzed by chemiluminescence (Pierce Chemical, Rockford, IL). Immunoblot data were quantified by AlphaEaseFC 4.0 software (Alpha Innotech Corp. San Leandro, CA).

### Immunoprecipitation

Cells were plated in 6-well plates and transfected overnight, as described above. The cell lysates were incubated with primary antibodies overnight at 4°C and then incubated with Protein A- or G- Sepharose (Sigma) for an additional 1 h at 4°C. The beads were washed three times with STE buffer (100 mM NaCl, 20 mM Tris-HCl, pH 7.5, 1 mM EDTA) and boiled in Laemmli sample buffer [13]. The immunoprecipitates were resolved by SDS-PAGE and analyzed by immunoblotting.

### TBC1D3 degradation assay

Cells were cultured in 12-well plates and transfected with Myc-TBC1D3. At 18 h after transfection, the cells were starved in serum-free medium for 3 h and either left untreated as a control or incubated with 10% fetal calf serum (FCS). The cells were then washed with ice-cold PBS and lysed. The lysates were subjected to SDS-PAGE and immunoblotting was carried out with specific antibodies against TBC1D3 (2C7), GAPDH or tubulin.

### Ubiquitination Assay

TBC1D3 ubiquitination was detected in HeLa cells, under denaturing conditions. Cells were seeded in 6-well plates and transfected with HA-CUL7 and Myc-TBC1D3, as described above. The cells were serum-starved in the presence of MG132 (20 µM) for 6 hours, treated with 10% fetal calf serum (FCS) for 20 minutes at 37°C and lysed in SDS-containing lysis buffer (1%SDS, 50 mM Tris-HCl, pH 7.5, 100 mM NaCl, 1% Triton X-100, 10% glycerol, 1 mM EDTA, 10 mM N- ethylmaleimide and protease inhibitor cocktail supplemented with 10 mM NaF and 1 mM Na_3_VO_4_). The lysates were boiled for 5 min, diluted in 10 volumes of the lysis buffer without SDS, and subjected to immunoprecipitation and immunoblot analysis.

### Alkaline Phosphatase Treatment

Whole-cell lysates were prepared from GFP-TBC1D3-expressing cells. TBC1D3 was immunoprecipitated with rabbit anti-GFP antibody, incubated with protein G beads and treated with 1 unit of alkaline phosphatase (Roche) in dephosphorylation buffer (50 mM Tris-HCl, 0.1 mM EDTA, pH 8.5) at 37°C for 1 h. The treated TBC1D3 beads were washed in STE buffer and incubated with Myc-Fbw8 cytosol prepared from Myc-Fbw8-expressing cells (as described above). Following an overnight incubation at 4°C, the beads were washed, eluted with sample buffer, resolved by SDS-PAGE and analyzed by immunoblotting. For GST pull-down experiments, cell lysates prepared from GFP-TBC1D3 expressing cells were treated with or without 2 units of alkaline phosphatase in dephosphorylation buffer at 37°C for 1 h. The treated lysates were incubated with 10 µg GST or 2 µg GST-Fbw8-Skp1 fusion proteins coupled to glutathione-Sepharose beads. Proteins, pulled-down with GST-Fbw8-Skp1 or GST-control beads, were separated by SDS-PAGE and analyzed by immunoblotting, using a monoclonal anti-TBC1D3 antibody.

## Results

### TBC1D3 is ubiquitinated and degraded in response to GF signaling

**Figure 1:**
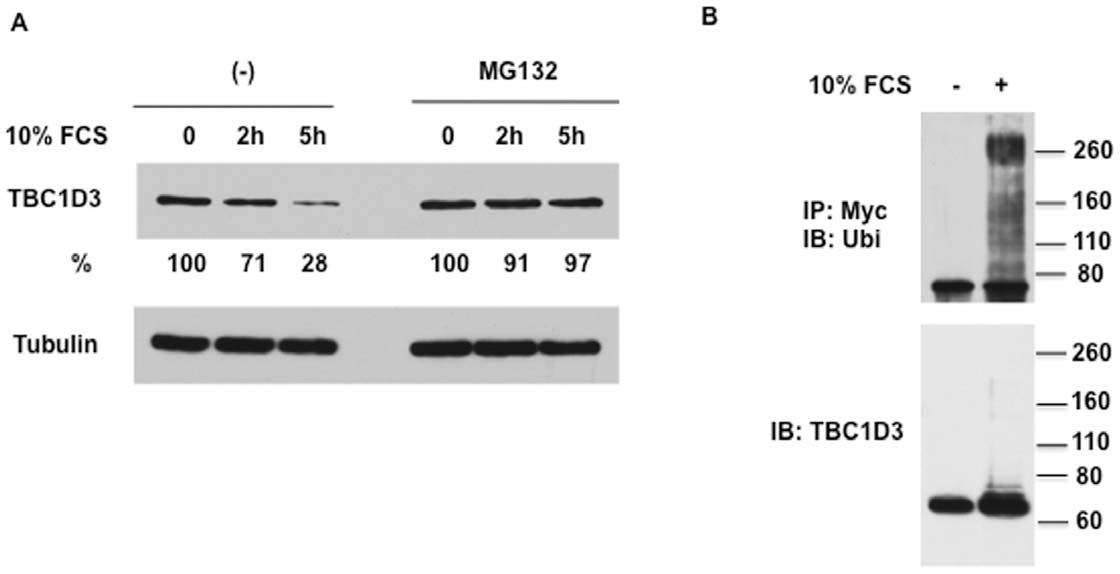
TBC1D3 is degraded and ubiquitinated. (A) TBC1D3 degradation is inhibited by MG132. HeLa cells were transfected with Myc-TBC1D3. The cells were starved in serum-free DMEM with or without MG132 (20 µM) at 37°C for 3 h and then stimulated with 10% FCS. For the MG132 treatment group, MG132 was kept in the medium until the cells were collected. Cell lysates were resolved by SDS-PAGE and immunoblotted with anti-TBC1D3 and tubulin antibodies. TBC1D3 was substantially degraded over the 5 h incubation period, which was blocked by MG132 treatment. The number below each time point blot records the percent remaining normalized to the zero time. (B) TBC1D3 is ubiquitinated. Hela cells transfected with Myc-TBC1D3, were starved with DMEM containing 20 µM MG132 for 6 h and stimulated with 10% FCS for 20 min. The cell lysates were prepared under denaturing conditions and Myc-TBC1D3 was immunoprecipitated with anti-Myc antibody, followed by immunoblotting with anti-ubiquitin antibody to visualize ubiquitinated TBC1D3 (top) or anti-TBC1D3 to visualize Myc-TBC1D3 (bottom). The migration of molecular weight standards is indicated. The experiments were repeated three times.

Previous work demonstrated that TBC1D3 enhanced the signaling and trafficking properties of the EGF and insulin receptors [9], [10]. To explore the role of enhanced GF signaling on TBC1D3 protein turnover we stimulated TBC1D3-expressing HeLa cells with different GFs as well as with fetal calf serum (FCS) as a general source of GFs. We found that stimulation with EGF, insulin or IGF-1 (data not shown) as well as stimulation by FCS induced TBC1D3 degradation (Figure 1A). We hereafter used FCS as a source of GFs in our experiments.

To explore the mode of degradation and the potential role of proteasomes, TBC1D3-transfected HeLa cells were serum-starved and then pulsed with FCS in the presence or absence of MG132, a proteasome inhibitor. Cycloheximide (CHX) (25 µg/ml) was used to block *de novo* TBC1D3 synthesis. MG132 blocked the degradation of TBC1D3 in response to FCS (Figure 1A), indicating that FCS stimulation promoted the proteasomal degradation of TBC1D3. Treating cells with NH_4_Cl to block lysosomal degradation had no effect on TBC1D3 degradation (data not shown). Moreover, when TBC1D3 degradation was blocked by MG132, we readily detected ubiquitinated TBC1D3 in FCS-stimulated cells (Figure 1B), suggesting that GF stimulation induces ubiquitination of TBC1D3.

### Identification of TBC1D3 binding partners

**Figure 2:**
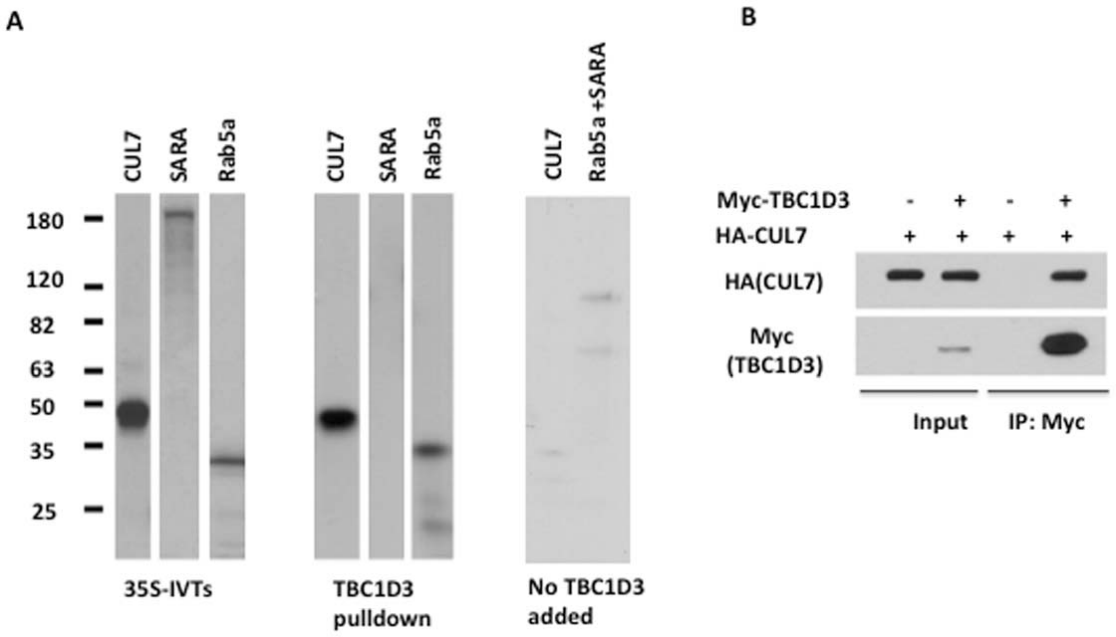
TBC1D3 interacts with CUL7. (A) *In vitro* transcription/translation assay. Plasmid inserts encoding putative TBC1D3 binding partners were transcribed and translated *in vitro* using a coupled, T7 polymerase-driven reaction with ^35^S-methionine. The SARA and Rab5a are the full-length human proteins while the CUL7 is an amino-terminal portion of the human CUL7. The proteins were resolved using SDS-PAGE, and TBC1D3 binding was visualized by autoradiography. The migration of molecular weight standards is indicated. (B) TBC1D3 co-immunoprecipitates with CUL7. HeLa cells were transfected with plasmids encoding HA-CUL7 and Myc-TBC1D3. Cells were lysed and immunoprecipitated with anti-Myc antibody. The immunoprecipitates were resolved by SDS-PAGE and blotted with anti-HA and anti-Myc antibodies.

We identified a panel of TBC1D3-interacting proteins using a yeast two-hybrid screen with full-length TBC1D3 as bait to interrogate a human fetal brain cDNA library (data not shown). *In vitro* transcription/translation assay was used to confirm the physical interaction between TBC1D3 and some of the TBC1D3-binding partners identified in the yeast two-hybrid screen. Plasmid inserts encoding amino-terminal portion of the human CUL7 (amino acids 239–487), as well as full length Rab5a and SARA (Smad Anchor for Receptor Activation), were transcribed and translated *in vitro*, using a ^35^S-methionine labeling system (Figure 2A, left panel).

The radiolabeled CUL7, Rab5a and SARA were incubated with TBC1D3-bound protein A beads (TBC1D3 pull-down) (Figure 2A, middle panel). Radiolabeled CUL7 or a mixture of Rab5a and SARA were incubated with bovine serum albumin (BSA)-bound protein A beads (no TBC1D3 added) as negative control (Figure 2A, right panel). The bound proteins were resolved using SDS-PAGE and visualized by autoradiography. TBC1D3 was able to efficiently pull-down CUL7 and Rab5a, but not SARA. The amino-terminal fragment of CUL7 (amino acids 239–487) identified in this screen, as a TBC1D3-binding partner contains the CPH domain that was previously implicated in protein-protein interaction and was shown to mediate the association of CUL7 with p53 [14]. These findings suggested a specific *in vitro* interaction between TBC1D3 and CUL7.

The interaction between CUL7 and TBC1D3 was further verified by a pull-down assay *in-vivo* (Figure 2B). HeLa cells were transfected with HA-CUL7 and with or without Myc-TBC1D3, lysed and TBC1D3 was immunoprecipitated using anti-Myc antibody. The bound proteins were analyzed by immunoblotting. The results presented in Figure 2B, clearly demonstrate that HA-CUL7 is efficiently pulled-down by Myc-TBC1D3, suggesting a specific *in vivo* interaction of TBC1D3 with CUL7. Collectively, these findings indicate that TBC1D3 and CUL7 are recruited into the same macromolecular complex through the direct interaction of TBC1D3 with the CPH domain-containing fragment of CUL7.

### GF-induced ubiquitination and degradation of TBC1D3 is CUL7-dependent

**Figure 3:**
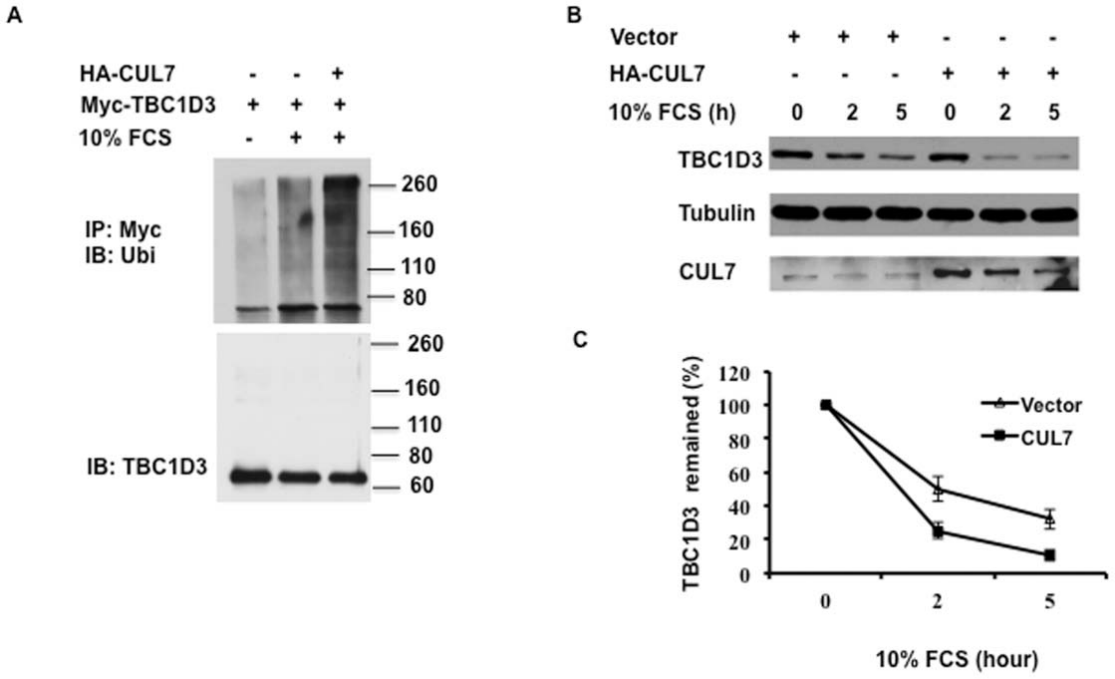
CUL7 mediates the degradation and ubiquitination of TBC1D3. (A) CUL7 increased TBC1D3 ubiquitination. HeLa cells were transfected with Myc-TBC1D3 and with or without HA-CUL7. TBC1D3 was immunoprecipitated with anti-Myc antibody. Polyubiquitination of TBC1D3 was analyzed by immunoblotting, using anti-ubiquitin antibody. (B) CUL7 expression enhances TBC1D3 degradation. HeLa cells were transfected with Myc-TBC1D3 and with or without HA-CUL7. Cells were starved for 3 h and stimulated with 10% FCS. Lysates were separated by SDS-PAGE and immunoblotted with anti- TBC1D3, CUL7 and tubulin antibodies. (C) Summary of results. Mean ± SE of 3 independent experiments. TBC1D3 expression is normalized by tubulin. The initial level of TBC1D3 expression in each group is set to 100%.

The specific interaction of TBC1D3 with CUL7 implies that CUL7 is involved in regulation of TBC1D3 ubiquitination and degradation. These were examined in HeLa cells transfected with Myc-TBC1D3 alone or together with HA-CUL7. The cells were starved for 6 h and stimulated with 10% FCS for 20 min at 37°C. TBC1D3 was immunoprecipitated under denaturing conditions with polyclonal anti-Myc antibody, resolved by SDS-PAGE, and analyzed by immunoblotting with anti-ubiquitin antibody. Introduction of HA-CUL7 into TBC1D3-expressing cells resulted in robust increase in TBC1D3 ubiquitination (Figure 3A), suggesting that CUL7 E3 ligase mediates TBC1D3 ubiquitination in HeLa cells.

To examine the effect of CUL7 expression on GF-induced TBC1D3 degradation, HeLa cells, transfected with Myc-TBC1D3 and vector alone or HA-CUL7, were starved and incubated with 10% FCS for the indicated times in the presence of cycloheximide (CHX) (Figure 3B, 3C). TBC1D3 levels were monitored over a 5-hour period. Overexpression of CUL7 strongly accelerated the FCS-induced degradation of TBC1D3, suggesting that CUL7 is involved in GF-induced TBC1D3 degradation.

**Figure 4:**
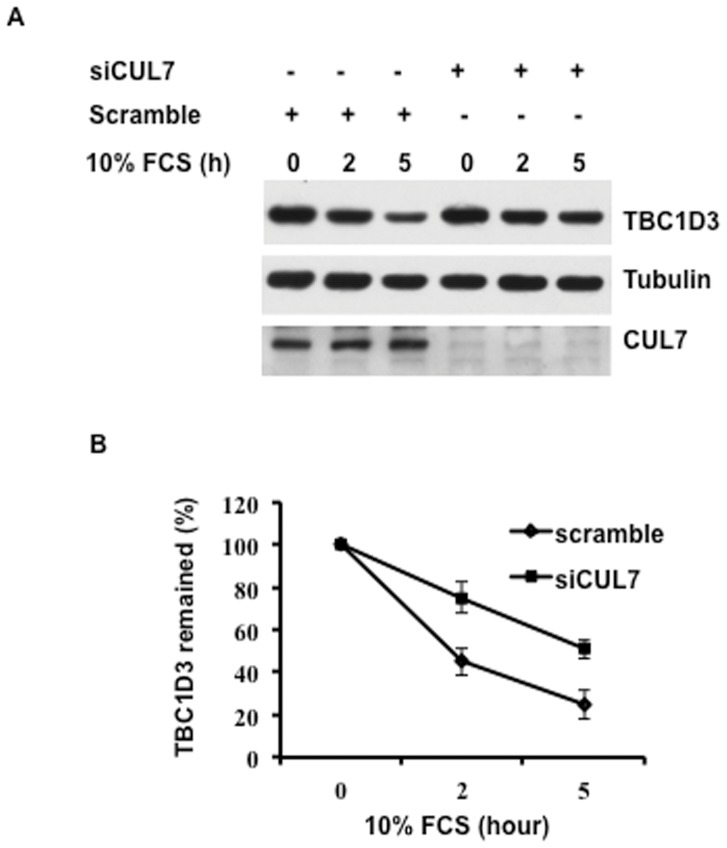
CUL7 siRNA silencing delays TBC1D3 degradation. (A) HeLa cells were transfected with scramble siRNA or CUL7 siRNA. At 24 h post-transfection, Myc-TBC1D3 was expressed in the cells for another 18 h. The cells were then starved for 3 h and stimulated with 10% FCS for different time periods. Cell lysates were prepared, separated by SDS-PAGE and immunoblotted with anti- TBC1D3, CUL7 and tubulin antibodies. (B) Summary of results. Mean ± SE of 3 independent experiments. TBC1D3 expression is normalized by tubulin. The initial level of TBC1D3 expression in each group is set to 100%.

To further verify the role of CUL7 in TBC1D3 degradation, HeLa cells were transfected with either CUL7-specific or scrambled small interfering RNAs (siRNAs). After starvation and stimulation with 10% FCS, cell lysates were prepared and CUL7 and TBC1D3 levels were analyzed by immunoblotting. The western blots shown in Figure 4A and quantified in Figure 4B indicate that CUL7 depletion delayed the degradation of TBC1D3, further implicating CUL7 in regulation of TBC1D3 degradation.

### Fbw8 is recruited to CUL7 TBC1D3 complex

**Figure 5:**
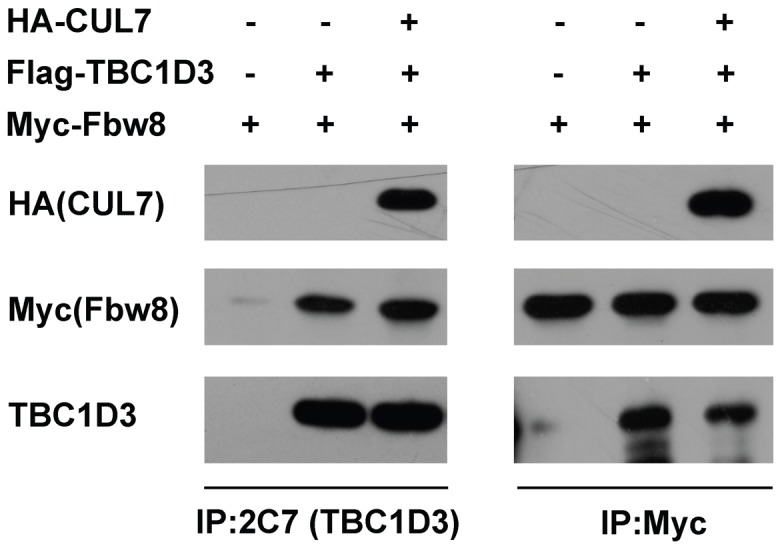
Interactions between expressed Fbw8, CUL7 and TBC1D3. HeLa cells were transfected with HA-CUL7, Flag-TBC1D3 and Myc-Fbw8 constructs. At 18 h after transfection, cell lysates (200 µg) were immunoprecipitated with anti-TBC1D3 (2C7) (left panel) or anti-Myc (right panel) antibodies, separated by SDS-PAGE and immunoblotted as indicated.

The CUL7 E3 ligase complex includes an adaptor protein, Skp1 and a substrate recognition protein Fbw8 that recognizes phosphorylated substrates [11], both tethered to the N-terminus of the CUL7 scaffold. To study the association of TBC1D3 with CUL7 and Fbw8, co-immunoprecipitation experiments were carried out in HeLa cells. As shown in Figure 5 (left panel), Flag-TBC1D3 is pulled down by Fbw8 when the two were co-expressed. When both Myc-Fbw8 and HA-CUL7 were co-expressed with Flag-TBC1D3, TBC1D3 pulled down both CUL7 and Fbw8. A similar pull-down experiment was carried out by Myc-Fbw8, Figure 5 (right panel). The control lane indicates that Protein G alone was unable to pull down TBC1D3. Immunoprecipitation with anti-Myc antibody pulled down TBC1D3 when TBC1D3 and Fbw8 were co-expressed, in the absence of CUL7, confirming an interaction between the proteins. When both Flag-TBC1D3 and HA-CUL7 were co-expressed with Myc-Fbw8, anti-Myc antibody pulled down both Flag-TBC1D3 and HA-CUL7. Together, these findings indicated that TBC1D3 interacts directly with the CUL7 scaffold as well as with CUL7 E3 ligase substrate recognition domain Fbw8.

### TBC1D3 and Fbw8 binding is phosphorylation-dependent

**Figure 6:**
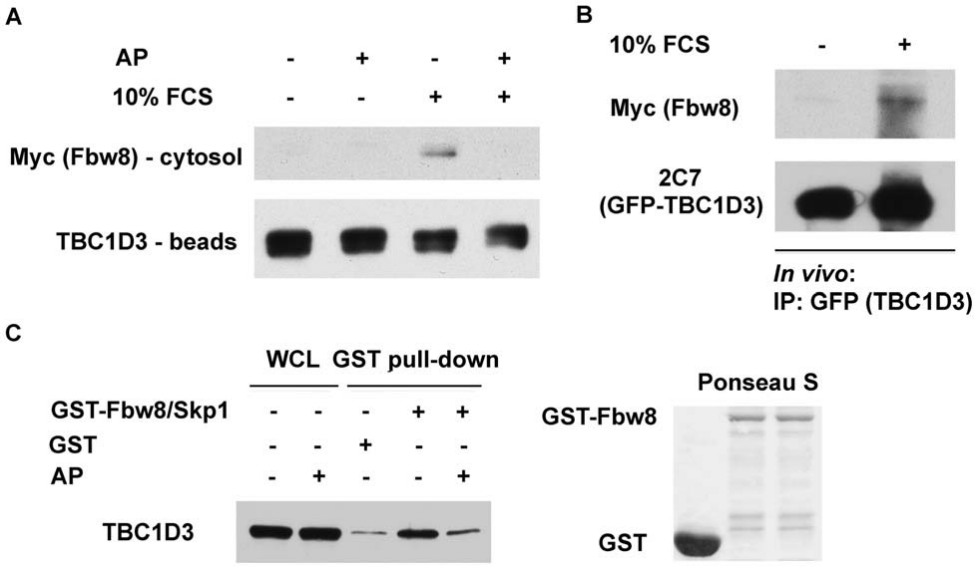
TBC1D3 interacts with Fbw8 in a phosphorylation-dependent manner. (A) *In vitro* interaction between TBC1D3 and Fbw8. Separate sets of HeLa cells were transfected with GFP-TBC1D3 or Myc-Fbw8. At 18 h after transfection, the GFP-TBC1D3-expressing cells were starved and stimulated with 10% FCS. To prepare TBC1D3-beads, GFP-TBC1D3 was immunoprecipitated with mouse anti-GFP antibody on Protein G beads. The TBC1D3-beads were treated with or without alkaline phosphatase (AP) for 1 h at 37°C. TBC1D3-beads were incubated with Fbw8-cytosol prepared from HeLa cells expressing Myc-Fbw8. Following incubation at 4°C, the beads were washed and the bound proteins were separated and analyzed by immunoblot using anti-Myc antibody. TBC1D3-beads pulled down Fbw8 and alkaline phosphatase treatment blocked TBC1D3-Fbw8 interaction. (B) *In vivo* interactions between TBC1D3 and Fbw8. HeLa cells were co-transfected with GFP-TBC1D3 and Myc-Fbw8. After 18 h, the cells were starved and stimulated with 10% FCS. Cell lysates were treated with and without AP and immunoprecipitated with anti-GFP antibody. The precipitates were separated by SDS-PAGE followed by immunoblot analysis. (C) GST-Fbw8-Skp1 pull-down of TBC1D3 is phosphorylation dependent. Glutathione beads with bound GST-Fbw8-Skp1 complex or GST alone were incubated with GFP-TBC1D3 expressing lysates, treated with or without AP for 1 hour at 37°C. The beads were washed and eluted proteins were separated by SDS-PAGE. Immunoblotting with monoclonal anti-TBC1D3 antibody (2C7) showed that GST-Fbw8-Skp1 pull-down of TBC1D3 was nearly abolished by prior alkaline phosphatase treatment (right panel). The right panel shows the Ponceau S staining of the transferred membrane. The experiments were repeated three times.

To determine whether TBC1D3 interacts with Fbw8 in a phosphorylation-dependent manner, an *in vitro* pull-down experiment was carried out with TBC1D3 bound to Protein-G beads (TBC1D3-beads) and a cytosol preparation obtained from a separate set of cells overexpressing Fbw8 (Fbw8-cytosol) (Figure 6A). The TBC1D3-beads were prepared from HeLa cells transfected with GFP-TBC1D3. After starvation, the cells were stimulated with or without FCS, lysed and TBC1D3 was immunoprecipitated with anti-GFP antibody. TBC1D3-beads were pre-incubated with or without alkaline phosphatase (AP) (37°C; 60 min). To test the recruitment of Fbw8 by TBC1D3, Fbw8-cytosol was added to each immunoprecipitate. The TBC1D3-beads were then washed and eluted with Laemmli sample buffer. Proteins associated with the anti-GFP pull-downs were then separated by SDS-PAGE and immunoblotted for Fbw8. Figure 6A shows that in the absence of FCS stimulation, there was little recruitment of Fbw8 by the TBC1D3-beads. However, TBC1D3-Fbw8 binding was detected following FCS stimulation, suggesting a GF-dependent interaction between TBC1D3 and Fbw8. Moreover, treatment of TBC1D3-beads with AP abolished the ability of Fbw8 to bind to TBC1D3. These findings suggested that FCS-induced binding of Fbw8 to TBC1D3 is phosphorylation-dependent.

A parallel experiment was carried out to complement this finding and to demonstrate that Fbw8 and TBC1D3 interaction is stimulated *in vivo* by GF stimulation. HeLa cells were co-transfected with GFP-TBC1D3 and Myc-Fbw8, starved and stimulated with FCS (Figure 6B). TBC1D3 was immunoprecipitated with anti-GFP antibody and the precipitates were probed for Fbw8 with anti-Myc antibody. The results presented in Figure 6B clearly demonstrate that TBC1D3 was able to pull-down Fbw8, only after stimulation with FCS, suggesting that Fbw8 association with TBC1D3 depends on GF stimulation (Figure 6B).

Lastly, the phosphorylation-dependent interaction between TBC1D3 and Fbw8 was examined using an *in vitro* GST pull-down assay. GST-Fbw8 is unstable when expressed alone in bacteria. However Pan and colleagues [15] showed that a GST-Skp1-Fbw8 construct is efficiently and stably expressed and is biologically active. Lysates prepared from TBC1D3-expressing cells were treated with or without AP and then incubated with GST or GST-Skp1-Fbw8 immobilized on glutathione beads. The input amounts of TBC1D3, GST and GST-Skp1-Fbw8 proteins before and after AP treatment were equivalent (Figure 6C, left panel, first two lanes and Figure 6C, right panel).

Incubation with GST alone resulted in some TBC1D3 binding which appeared to be non-specific (Figure 6C, left panel, lane 3). Importantly, incubation with GST-Skp1-Fbw8 resulted in a pronounced increase in TBC1D3 binding (Figure 6C, left panel, lane 4), which was largely abolished by AP treatment (Figure 6C, left panel, lane 5).

### Interaction between CUL7 and Fbw8 is required for TBC1D3 degradation

**Figure 7:**
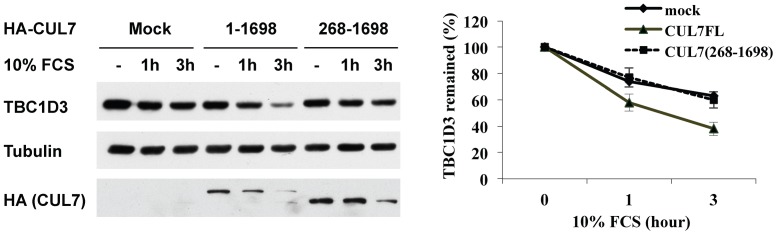
CUL7-mediated TBC1D3 degradation requires a CUL7-associated Fbw8. HeLa cells were transiently transfected with Myc-TBC1D3 along with mock, full-length CUL7 (1–1698) or truncated CUL7-mutant (268–1698) that is unable to recruit Fbw8. The cells were starved and stimulated with 10% FCS for different times. Lysates were resolved by SDS-PAGE at the indicated times. Immunoblotting was carried out with anti-Myc, -tubulin and -HA antibodies (left panel). Right panel shows mean ± SE of 3 independent experiments. TBC1D3 expression is normalized by tubulin. The initial level of TBC1D3 expression in each group is set to 100%.

Previous studies have shown that an N-terminal truncated mutant of CUL7 (CUL7: 268–1698) failed to bind Fbw8, thus preventing the assembly of a functional E3 ligase [3]. To determine whether CUL7-mediated TBC1D3 degradation required the associated E3 ligase activity, full-length and a N-terminal truncated mutant of CUL7 were co-expressed with Myc-TBC1D3 in HeLa cells. Following stimulation with FCS, TBC1D3 degradation rates were measured by immunoblot analysis. As shown in Figure 7, expression of full-length CUL7 (1–1698) accelerated TBC1D3 degradation. In contrast, the Fbw8-binding-deficient mutant, CUL7: 268–1698 was ineffective in increasing TBC1D3 degradation. We conclude that CUL7 binding to Fbw8 is required for TBC1D3 degradation.

## Discussion

Delineating the evolution and function of hominoid- and human-specific genes may open a window critical to understanding human physiology and disease. *TBC1D3* arose as recently as 25 million years ago within the primate lineage and has since been amplified by segmental duplication [16]. The human genome encodes as many as 53 copies of TBC1D3 [17] while our nearest primate neighbor, the chimpanzee genome, encodes a single copy [18]. The positive selection that generates the relative enrichment of *TBC1D3* in the human genome might be driven by an important biological function of TBC1D3 protein, however the exact role of TBC1D3 in human physiology is still unclear.

TBC1D3 is overexpressed or mutated in a variety of human cancers [2], [3], [4], [5], implying its involvement in tumor development and/or progression. Notably, several reports indicated that TBC1D3 expression enhanced the activation of GF-receptor signaling [8], [9], [10], indicating that unrestrained expression of TBC1D3 would result in enhanced cell growth and proliferation. In agreement with that, ectopic expression of TBC1D3 in murine fibroblasts was able to induce transformation of the cells, resulting in increased cell proliferation and uncontrolled growth [2].

While studying the biological effects of TBC1D3 in cultured cells, we observed that stimulation of cells with a variety of GFs (or with fetal calf serum) led to TBC1D3 degradation. This finding prompted us to examine the factors that regulate degradation of TBC1D3. Our yeast two-hybrid screen with TBC1D3 as the bait and human fetal brain library as the prey identified a small number of hits, one of which was CUL7– a molecular scaffold that facilitates the assembly of the CUL7 E3 ligase complex.

Substrate recruitment to the E3 complex may require participation of multiple E3 components. To demonstrate that each component of the CUL7 complex, viz., the CUL7 scaffold and the Fbw8 recognition component, plays a role in the ubiquitination and degradation of TBC1D3, we tested each protein separately.

We applied several experimental approaches to study the interaction between TBC1D3 and CUL7 and confirmed the direct association of TBC1D3 with CUL7 both *in vitro* and *in vivo*. Furthermore, overexpression or suppression of CUL7 increased or decreased, respectively the degradation of TBC1D3 in response to GF stimulation, confirming the role of CUL7 in TBC1D3 degradation.

We then tested the effect of GF stimulation on Fbw8 association with TBC1D3. Using TBC1D3 prepared from cells stimulated with fetal calf serum, we were able to recruit Fbw8 from a cytosol preparation where Fbw8 was overexpressed. Alkaline phosphatase treatment abolished the ability of TBC1D3 to recruit Fbw8, suggesting that GF-induced association of TBC1D3 and Fbw8 is phosphorylation-dependent.

Lastly, using a bacterially produced GST-Fbw8, we were able to pull down TBC1D3 prepared from GF-stimulated cells. Moreover, the interaction between TBC1D3 and Fbw8 was abolished by alkaline phosphatase treatment of the TBC1D3 preparation, suggesting again that this interaction is phosphorylation-dependent.

CUL7 is unique among the Cullin family of proteins. It is larger than other Cullin family members and unlike the other Cullins, CUL7 is known to bind a limited number of important proteins including P53, CUL1, PARC and T antigen [11], [19]. P53 and T antigen may recruit CUL7 to functional complexes that operate in the cytoplasm or the nucleus [20]. We documented here that TBC1D3 binds to CUL7 through the same region as P53 [14]. This region contains the CPH domain, a domain implicated in protein-protein interactions [14]. CUL7 is also unique amongst its family members in that it interacts with a single F-box substrate recognition unit, Fbw8. Substrates that are recognized and ubiquitinated by CUL7-Fbw8 are few in number but include IRS-1 [15], Cyclin D1 [21], GRASP65 [22] and now, TBC1D3.

Recent work with a short stature syndrome, called 3 M Syndrome, has linked CUL7 to growth in humans [23]. Interestingly, 3 M Syndrome is precipitated by mutations not only in CUL7 but also in OBSL1 and CCDC8, two proteins that associate with CUL7 [24]. OBSL1 is reported to interact directly or indirectly with the C-terminus of CUL7 and to recruit it to the Golgi complex of neurons where it regulates and ubiquitinates GRASP65, a regulator of Golgi function [22]. Whether there is any connection between OBSL1 recruitment of CUL7 to the Golgi and growth retardation in 3 M Syndrome is not known. However, the recruitment of CUL7 to an intracellular target by OBSL1, a protein apparently operating as an adaptor, raises the possibility that TBC1D3, by virtue of its direct interaction with CUL7 may play a role in recruiting CUL7 to a macromolecular complex (perhaps similar to that proposed for T antigen or P53) that regulates GF signaling.

CUL7 has been shown to ubiquitinate IRS-1 as part of the negative feedback mechanism to suppress insulin signaling [15]. IRS-1 is phosphorylated on sites required for degradation by p70 S6 kinase, a key enzyme in the mTOR pathway. Our recent work shows that TBC1D3 suppresses IRS-1 phosphorylation, ubiquitination and degradation by selectively inactivating S6 kinase [10]. TBC1D3 interacts with the PP2A subunit B56γ a regulatory subunit known to dephosphorylate and inactivate S6 kinase [25]. This results in delayed IRS-1 degradation and enhanced insulin signaling. We speculate that TBC1D3 may recruit CUL7 into a macromolecular complex, perhaps with PP2A:B56γ, where it has an effect on GF signaling and growth while simultaneously orchestrating TBC1D3 ubiquitination and degradation.

**Figure 8:**
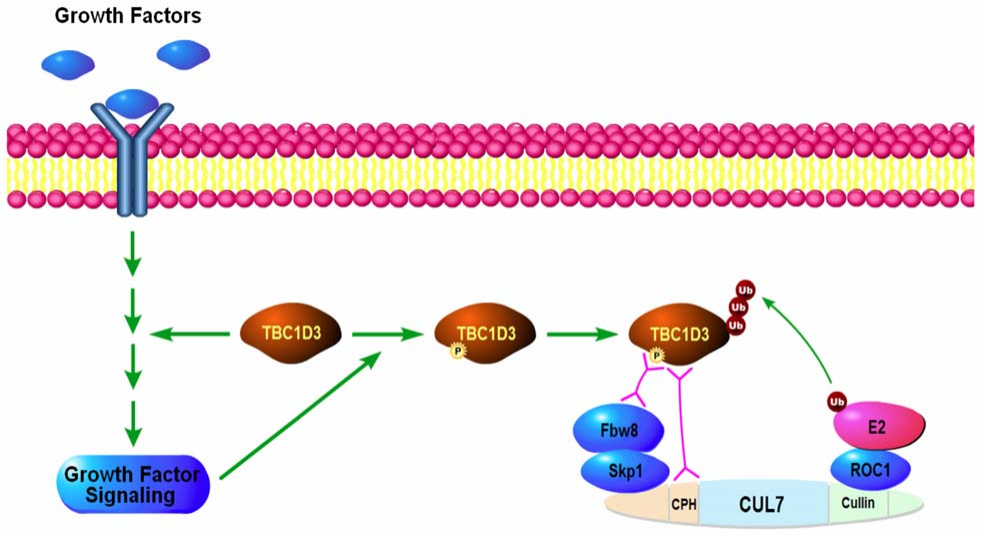
The proposed model for CUL7-mediated TBC1D3 degradation. TBC1D3 facilitates the propagation of GF-receptor signaling. In turn, GF stimulation induces TBC1D3 phosphorylation and phosphorylated TBC1D3 is recruited to Fbw8. CUL7 provides the structural backbone for the assembly of the CUL7-E3 ligase. The N-terminal portion of CUL7 binds to the adapter protein Skp1 that in turn binds the substrate recognition component, Fbw8. The RING E3 ligase associated with the C-terminus of CUL7 is called RBX1 or ROC1. It recruits an E2 ligase that provides the charged ubiquitin for transfer to the Fbw8-bound TBC1D3. TBC1D3 binds to CUL7 directly through the CPH domain as well as to Fbw8. TBC1D3 is degraded by CUL7-E3 ligase. This creates an inactivation loop that suppresses the effect of TBC1D3 on GF-receptor signaling. TBC1D3 binding to the CPH domain may also allow TBC1D3 to recruit CUL7 to unknown regulatory complexes where additional CUL7 substrates could be more efficiently ubiquitinated.

In Figure 8, we offer a speculative model that summarizes our findings and identifies new directions. TBC1D3 enhances activation of GF-receptor signaling, by a yet unknown mechanism. Upon signal activation, we propose that TBC1D3 is phosphorylated by unknown kinases. TBC1D3 phosphorylation leads to the assembly of a functional macromolecular complex via TBC1D3 interaction with the CUL7 scaffold, through the CPH-domain-containing region of CUL7. Interaction of TBC1D3 with the CUL7 scaffold facilitates its recognition by Fbw8 followed by ubiquitination and degradation. TBC1D3 degradation suppresses the TBC1D3-induced activation of the GF receptor signaling. Hence, GF-receptor signaling creates an inactivation loop that limits the effect of TBC1D3 on signal transduction. The molecular mechanisms of TBC1D3-induced activation of GF-receptor signaling as well as the identity of kinases that might mediate GF-induced phosphorylation of TBC1D3 will need to be identified in the future studies.

Our findings provide novel insights into the regulatory mechanisms of TBC1D3 protein turnover. Putatively, these mechanisms might be dysregulated in human tumors, resulting in unrestrained TBC1D3 expression, increased GF-receptor signaling and cellular transformation. Future studies will address the mechanisms that regulate TBC1D3 expression and degradation in human tumors.
